# Giant urethral diverticulum's caused by traumatic vesicle catheterization in children: a case report

**DOI:** 10.4314/pamj.v10i0.72224

**Published:** 2011-10-10

**Authors:** Khalid Kkhattala, Mohamed Rami, Aziz Elmadi, Lamia Chater, Abdelhalim Mahmoudi, Youssef Bouabadallah

**Affiliations:** 1 Department of Paediatric Surgery, University hospital of Fez, Fez, Morocco

**Keywords:** Giant, urethral diverticulum, child, surgery, urology, traumatism

## Abstract

Urethral diverticula are saclike dilations of the urethra and are classified as either congenital or acquired. While urethral diverticula are commonly seen in female patients, they are rarely seen in men. The most common etiologies of male acquired diverticula include urethral trauma, stricture, abscess or post-hypospadias repair. We report a case of acquired urethral diverticula caused by a traumatic vesical catheterization in a 6-year old boy and review the literature on the topic.

## Introduction

Male urethral diverticula are rare, they are classified as either congenital or acquired. The authors report a case of urethral diverticulum secondary to an exceptional aetiology in a 6 years old boy.

## Patient and case report

**Figure 1:**
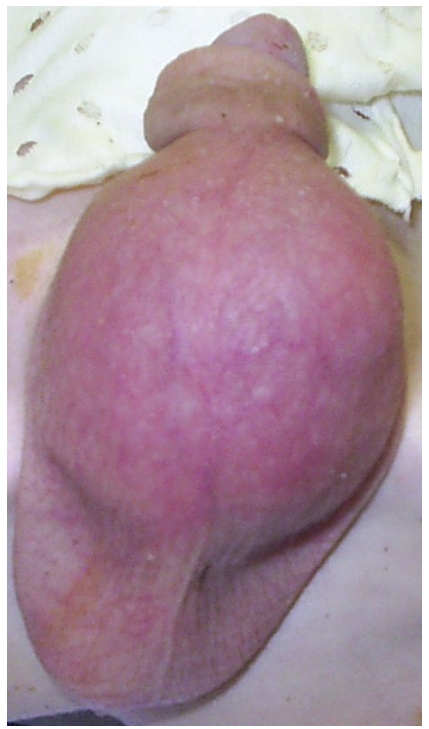
Mass on the ventral root of the penis of six years old boy who developed a large urethral diverticulum after vesical catheterization

**Figure 2:**
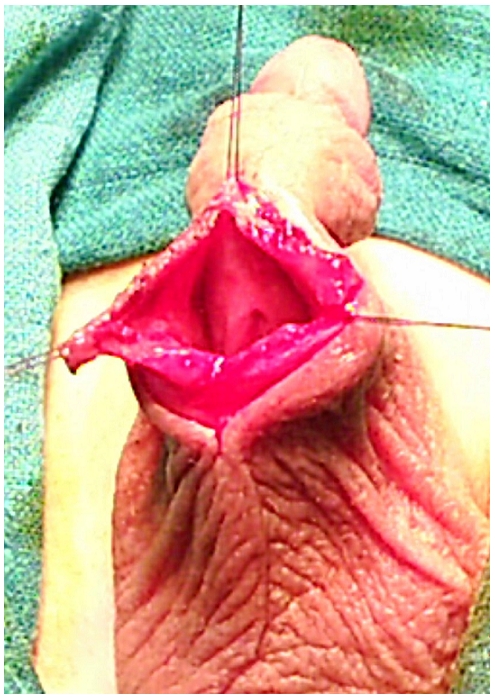
The urethral diverticulum opened

**Figure 3:**
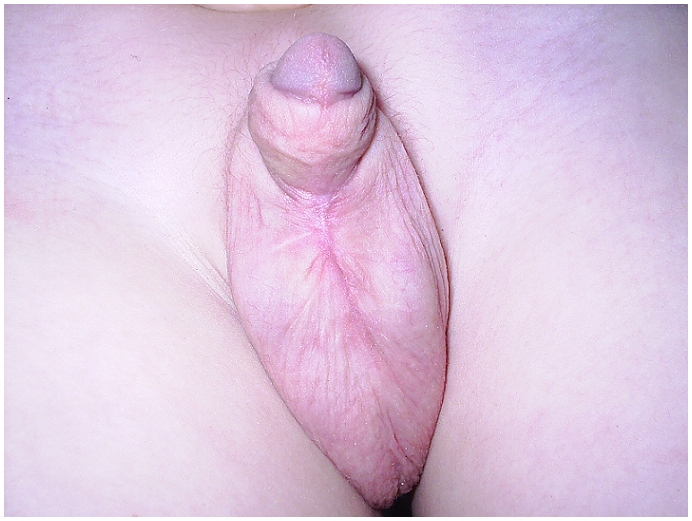
Post urethral surgery aspect in a 6-years old boy who developed a large diverticulum after vesical catheterization

A 6-years old boy was hospitalized in intensive care unit for severe cranial trauma. The patient developed a large diverticulum after vesical catheterization. It manifested as a mass on the ventral root of the penis (Figure 1) which caused urinary discharges when pressed gently. This mass increased gradually, leading to a penoscrotal fistula. A cystostomy was carried out, the diverticulum was opened to drawn the abscess (Figure 2). Five months later, we performed uretroplasty. The post surgery follow up was simple; the evolution was favourable at 5 years postop (Figure 3). The patient keeps a hemiparesy allowing walk.

## Discussion

Male urethral diverticula are rare. Acquired diverticula are only slightly more frequent than congenital ones. In 50% of these cases peri-urethral suppuration was the promoting etiology (other etiologies are: anterior urethral valves in children [1,2] and ectopic ureter). All were found to have a posterior urethral diverticulum thought to represent the remains of the original recto-urethral fistula accompanying the high rectal atresia [4].

Symptoms included post-micturition dribble, recurrent urinary infections, poor urinary stream and palpable swelling. The anterior urethra and peno-scrotal junction were the most commonly affected sites. Complications included urethra-cutaneous fistula [5], urethral stricture [2] and wound infection [4].

The diagnosis is clinical, based on the development of a perineal mass or phlegmon. This diagnosis is confirmed by cysto-urethrography. Treatment is by open surgery and consists on diverticulectomy and repair of urethra in one stage.

We recommend urethral diverticulectomy as the treatment of choice whenever possible. We stress prophylaxis via gentle manipulation of the urethra and proper drainage of peri-urethral suppuration.

## Conclusion

Urethral diverticula are uncommon, but should always be considered in young men with history of urethral surgery or trauma. The treatment is simple and the prognosis is usually good.
